# Effectiveness of Cognitive Orientation to daily Occupational Performance for autistic children with developmental coordination disorder

**DOI:** 10.1111/dmcn.16058

**Published:** 2024-08-14

**Authors:** Melika Kangarani‐Farahani, Sandy Thompson‐Hodgetts, Jill G. Zwicker

**Affiliations:** ^1^ Graduate Programs in Rehabilitation Sciences University of British Columbia Vancouver Canada; ^2^ Brain, Behaviour, & Development Theme BC Children's Hospital Research Institute Vancouver Canada; ^3^ Department of Occupational Therapy, Faculty of Rehabilitation Medicine University of Alberta Edmonton Canada; ^4^ Department of Occupational Science & Occupational Therapy University of British Columbia Vancouver Canada; ^5^ Department of Pediatrics (Division of Developmental Pediatrics) University of British Columbia Vancouver Canada

## Abstract

**Aim:**

To determine if the Cognitive Orientation to daily Occupational Performance (CO‐OP) intervention is effective in improving motor skills of autistic children with developmental coordination disorder (DCD), and whether motor gains are maintained 3 months after therapy.

**Method:**

In this quasi‐experimental study, we recruited 27 autistic children (8–12 years) with DCD without intellectual disability. The treatment group received CO‐OP intervention once weekly for 10 weeks, focusing on three child‐chosen motor goals. The waitlist group received CO‐OP 3 months later. Outcome measures included the Canadian Occupational Performance Measure (child ratings of motor performance and satisfaction), Performance Quality Rating Scale (therapist‐observed movement quality), and the Bruninks‐Oseretsky Test of Motor Proficiency, Second Edition (motor ability).

**Results:**

Non‐parametric analyses showed significant improvements (*p* < 0.013) in all outcomes. Follow‐up analysis revealed significant improvements in performance (*p* < 0.001, W = 0.69), satisfaction (*p* < 0.001, W = 0.72), and movement quality (*p* < 0.001, W = 0.62). Despite slight declines at follow‐up, overall improvements from pretest remained evident.

**Interpretation:**

The CO‐OP intervention effectively improved motor skills of autistic children.

## Graphical abstract

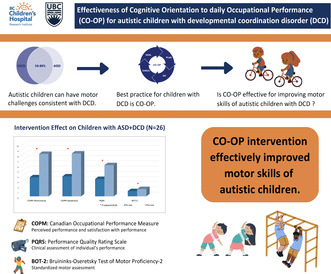

This original article is commented by Madieu and Cantin on pages 144–145 of this issue.

**Abbreviations**
ADHD: attention‐deficit/hyperactivity disorder
ASD: autism spectrum disorder
BOT‐2: Bruininks‐Oseretsky Test of Motor Proficiency, Second Edition
CO‐OP: Cognitive Orientation to daily Occupational Performance
COPM: Canadian Occupational Performance Measure
DCD: developmental coordination disorder
DCDQ: Developmental Coordination Disorder Questionnaire
MABC‐2: Movement Assessment Battery for Children, Second Edition
PQRS: Performance Quality Rating Scale
RCT: randomized waitlist‐control trial

What this paper adds

- Autistic children with developmental coordination disorder can learn motor skills with the Cognitive Orientation to daily Occupational Performance (CO‐OP) intervention.
- Improvements in functional goals, movement quality, and motor ability were observed after CO‐OP.
- Functional motor goals and movement quality were maintained 3 months after intervention.

Autism spectrum disorder (ASD) is a highly prevalent neurodevelopmental disorder characterized by challenges in social interactions, communication skills, and the presence of restricted and repetitive behaviors.^1^ While motor impairments are not included in the diagnostic criteria for ASD, research has indicated that motor deficits are present in 50% to 88% of children diagnosed with ASD.^2^ These findings align with the results of other studies that have confirmed a high occurrence of motor impairments in autistic children, which are frequently under‐diagnosed and under‐recognized.^3^, ^4^, ^5^ The motor deficits observed in autistic children (without intellectual disability) are consistent with developmental coordination disorder (DCD), a neurodevelopmental disorder that significantly impacts a child's ability to perform and learn motor skills.^1^ Since the publication of the Fifth Edition of the Diagnostic and Statistical Manual (DSM‐5) in 2013, a dual diagnosis of ASD and DCD has been permitted.^1^

The development of motor skills during childhood is crucial for engaging in more advanced and complex activities, including self‐care, leisure pursuits, social communication, and learning.^6^ Motor impairments in autistic children have significant consequences across various domains of their lives. For instance, fine motor deficits in autistic children contribute to poorer participation both at home and in school settings.^6^, ^7^ Additionally, the presence of gross motor deficits negatively affects their social interaction, engagement in leisure activities, and overall school experiences.^2^, ^3^, ^4^, ^5^, ^6^, ^7^ The poor motor performance of autistic children not only impacts their functional abilities, but also has significant long‐term effects on their social skills development, self‐esteem, adaptive function, and overall quality of life.^2^, ^8^, ^9^

Therapy for autistic children typically focuses on developing social and communication skills, as well as managing sensory processing differences.^10^, ^11^ Despite the high prevalence of motor impairments in autistic children, motor concerns are not typically the primary target of therapy for these children.^2^

Current best‐practice to improve motor function in children with DCD includes a treatment approach called Cognitive Orientation to daily Occupational Performance (CO‐OP).^12^ CO‐OP is a task‐specific approach designed to improve motor‐based skills that a child needs or wants to master; it is a cognitive‐based, problem‐solving approach that uses verbal mediation and identifies strategies to support skill acquisition.^13^

Results from a recent study have shown that children with DCD, as well as those with co‐occurring DCD and attention‐deficit/hyperactivity disorder (ADHD), experience improved motor performance with CO‐OP intervention.^14^ Preliminary findings from a case study of two autistic children suggest that CO‐OP is feasible and capable of inducing clinically significant improvements in self‐ and parent‐rated performance on their motor‐based occupational performance goals (hereafter called ‘motor goals’).^15^ However, it remains largely unknown whether autistic children with DCD also benefit from CO‐OP.

Thus, the objectives of this study were to determine: (1) the effectiveness of the CO‐OP intervention in enhancing self‐rated performance and satisfaction of functional motor goals, therapist‐rated motor quality, and overall motor skills; and (2) whether the outcomes are sustained 3 months after the intervention in autistic children with DCD. We hypothesized that autistic children with DCD would show statistically significant and clinically meaningful improvements in motor function after the CO‐OP intervention, and that they would maintain motor performance after 3 months. We did not anticipate a significant impact on motor proficiency as measured by the Bruininks‐Oseretsky Test of Motor Proficiency, Second Edition (BOT‐2) after the intervention.

## METHOD

### Study design

This study reports behavioral results of a registered randomized waitlist‐control trial (RCT) (ClinicalTrials.gov ID: NCT04119492) evaluating rehabilitation to improve outcomes for autistic children with DCD. Because of the smaller than anticipated sample size, we combined the treatment group and waitlist group, making this study a quasi‐experimental pretest‐posttest design.

### Sample size calculation

We determined that a sample of 30 autistic children with DCD would provide more than 80% power to detect clinically significant improvement of 2 points on the Canadian Occupational Performance Measure (COPM)^16^ with a standard deviation of 2.5 and a type‐1 error of 0.05.

### Participants

From February 2019 to July 2023, 27 autistic children with DCD (8–12 years) were recruited in the Greater Vancouver area, Canada from the DCD Clinic at Sunny Hill Health Centre for Children, the British Columbia Autism Assessment Network, and from advertisements in the community. Children were able to communicate verbally and met the diagnostic criteria for DCD as outlined in DSM‐5^1^ and international clinical practice guidelines:^12^ (1) a score of the 16th percentile or lower on Movement Assessment Battery for Children, Second Edition (MABC‐2);^17^ (2) an age‐dependant total score of 15 to 57 on the Developmental Coordination Disorder Questionnaire (DCDQ);^18^ (3) parent report of motor difficulties from a young age; and (4) having no other medical condition that could explain the motor deficits (e.g. cerebral palsy, intellectual disability) as per parent report, clinical observations, and/or medical exam. All children had a formal ASD diagnosis. We also used the Autism Spectrum Screening Questionnaire, a parent report questionnaire designed to screen autistic children with high or normal IQ (or those with only mild intellectual disability);^19^ a score of 19 and above indicates probable ASD, so children who scored below 19 were excluded. Because of the high co‐occurrence of ADHD with both ASD and DCD,^20^ children with ADHD were not excluded from participating in the study.

Participants were enrolled in the study after parental consent and child assent as per ethics approval from the Children's Women's Health Centre/University of British Columbia Research Ethics Board (#H18‐02759).

### Procedure

Children were randomly assigned to either a treatment or a waitlist group. A statistician randomized participants using computer‐generated sequential blocks of 4 to 6; randomization codes were kept in sealed opaque envelopes until study enrollment.

After enrollment, children in the treatment group participated in baseline motor assessments at BC Children's Hospital, followed by 10 weeks of CO‐OP, with each weekly session lasting 1 hour. Therapists kept detailed notes of strategies used for each goal, providing evidence of treatment fidelity. Post‐assessment was done within 1 to 2 weeks of completion of intervention, and reassessment was conducted 3 months after intervention. Children in the waitlist group waited 3 months after enrollment before completing the baseline motor assessments, 10‐week intervention, and post‐assessment. Figure S1 is a schematic of the design study.

All pre‐ and post‐assessments were conducted by an occupational therapist who was not involved in the intervention. The therapist first administered the Pediatric Activity Card Sort^20^ to assist the children in choosing three functional motor goals that were personally meaningful (e.g. tying shoes, riding a bike, printing legibly). The Pediatric Activity Card Sort is an occupation‐based assessment tool that consists of 75 cards describing childhood activities in different categories (i.e., personal care, school/productivity, hobbies/social activities, and sport).^20^ Table S1 shows the motor goals selected by the children. The COPM^21^ was used to measure the child's rating of their motor performance and satisfaction of their motor goals, the Performance Quality Rating Scale (PQRS) assessed movement quality,^22^ and BOT‐2^23^ measured general motor ability before and after intervention. All screening measures were ordinal except for the DCDQ, which was continuous.

### Measures

#### Screening measures

##### Movement Assessment Battery for Children, Second Edition

The MABC‐2 is a valid and reliable test that assesses fine and gross motor skills in 3‐ to 16‐year‐old children in three domains: manual dexterity, ball skills, and balance.^16^ Raw scores are converted to age‐specific normative percentile scores (ranging from 1st to 99th percentile); lower scores indicate greater motor impairment. According to international clinical practice guidelines, a cut‐off score of the 16th percentile or lower is used to meet diagnostic criterion A of DCD.^12^

##### Developmental Coordination Disorder Questionnaire

The DCDQ is a 15‐item parent questionnaire recommended by international clinical practice guidelines for screening motor impairment in children 5 to 15 years of age.^12^ Parents are asked to evaluate the child's performance in various activities of daily living compared to typically developing peers. The DCDQ has a high internal consistency, as well as 85% sensitivity and 71% specificity.^17^ The total score ranges from 15 to 75, with lower scores indicating poorer motor function.^12^

##### Autism Spectrum Screening Questionnaire

The Autism Spectrum Screening Questionnaire is a widely used screening questionnaire for children 6 to 17 years of age and is completed by parents or teachers.^18^ Parents completed the questionnaire for this study. This tool is intended as an initial screening measure for ASD, specifically in individuals with high or normal IQ, or those with mild intellectual disability. The results yield a total score ranging from 0 to 54, where higher scores suggest a greater presence of ASD characteristics. A score of 19 and above is indicative of probable ASD.^18^

##### Conners 3 ADHD Index

The Conners 3 ADHD Index—Parent form was used to assess ADHD symptomology.^24^ The Conners 3 ADHD Index is a 10‐item scale that assesses the presence of the most prominent symptoms of ADHD over the last month in children between 6 years and 18 years. A score of 70 or more indicates clinically significant ADHD symptomology (total score ranges from 0 to 90).

#### Outcome measures

##### Canadian Occupational Performance Measure

Our primary outcome measure was the COPM, which is an individualized, client‐centred measure designed to detect change in a client's self‐perception of their performance and satisfaction over time on functional goals that are of importance to them.^21^ The COPM is a 10‐point scale, with higher scores indicating greater performance and satisfaction. While a change of 2 or more points has been considered clinically significant on this measure for decades,^21^ recent evidence suggests other ways, such as the distribution method (dividing standard deviation by the square root of sample *n*), be used to calculate clinical significance.^25^

##### Performance Quality Rating Scale

The PQRS is an objective and observational measure of performance quality with moderate to substantial inter‐rater reliability, high test–retest reliability, and good internal responsiveness.^26^ To supplement the COPM, we recorded the child performing their three motor goals before and after intervention. The motor performance was scored by an occupational therapist who was not involved in the intervention and who was blinded to group allocation and pretest/posttest status. The PQRS uses a 10‐point scale; a change score of 3 points is considered clinically meaningful, however the smallest real difference scores for children range from 2.13 to 2.91.^22^ Therefore, an increase of 2.13 points can also be regarded as clinically significant.^22^

##### Bruininks‐Oseretsky Test of Motor Proficiency, Second Edition

The short form of the BOT‐2 was used to measure fine and gross motor skills. The BOT‐2 assesses children's motor proficiency in fine manual control, manual coordination, body coordination, and strength/agility.^23^ Raw scores are converted into standard scores and percentile ranks (range from 1st to 99th percentile); lower percentile scores indicate poorer motor ability. The BOT‐2 has excellent reliability and validity.^27^

### Data analysis

To investigate the effectiveness of the CO‐OP intervention for autistic children with DCD, we used pretest‐posttest analyses. The data collected before and after the intervention for this study were combined for both the waitlist and treatment groups to enhance statistical power, thereby making this a quasi‐experimental study rather than the intended RCT. However, the pre‐post analysis for each group separately was also conducted, and results are in Table S2. Statistical analyses were conducted using IBM SPSS Statistics version 23 (IBM Corp., Armonk, NY, USA). The normality of continuous variables was verified with the Shapiro–Wilk test. Table 1 presents the mean and standard deviations of these variables, along with the median and interquartile range for ordinal variables.

**TABLE 1:** Participant characteristics.

Variables	Treatment (*n* = 13)	Waitlist (*n* = 13)	*p*
Male sex assigned at birth; *n* (%)	10 (77)	11 (85)	0.62
Age (years:months); mean (SD)	9:6 (1:0)	10:6 (1:8)	0.14
DCDQ (total); mean (SD)	31.8 (8.6)	29.6 (6.3)	0.61
MABC‐2 (percentile); median (IQR)	1 (3.2)	1 (6.5)	0.62
Conner's ADHD Index (t‐score); median (IQR)	90 (9)	90 (5)	0.82
ASSQ (total); median (IQR)	27 (13)	24 (13)	0.80

Abbreviations: ADHD, attention‐deficit/hyperactivity disorder; ASSQ, Autism Spectrum Screening Questionnaire; DCDQ, Developmental Coordination Disorder Questionnaire; IQR, interquartile range; MABC‐2, Movement Assessment Battery for Children, Second Edition.

As all outcome measures were ordinal (COPM and PQRS using Likert scales and BOT‐2 reporting percentile scores), non‐parametric statistical tests were employed to address our research questions. The Wilcoxon signed‐rank test was utilized for pretest‐posttest comparisons for all outcome measures, including r‐value effect sizes. In order to evaluate the effectiveness of the treatment 3 months after the intervention, the non‐parametric Friedman test was employed to analyze and compare the pre‐intervention, post‐intervention, and follow‐up assessments within the treatment group. The Kendall's W test value was calculated for the Friedman test as a measure of effect. In all analyses, the alpha level was set at 0.05, with Bonferroni correction for multiple comparisons. Effect sizes for r‐values were interpreted using the following thresholds: small (0.2), moderate (0.5), and large (0.8).^28^ Effect sizes for W‐values were interpreted as small (0.147), medium (0.33), and large (0.474).^28^

## RESULTS

### Participants

As shown in Figure S2, the Consolidated Standards of Reporting Trials flowchart, from a total of 27 children recruited for this study, 26 autistic children with DCD (21 males, five females; mean [SD] age: 10 years [1 year 6 months]) participated in this study: 13 children were randomized into the treatment group and 13 children into the waitlist group. Participant characteristics including age, sex, and clinical assessment scores are described in Table 1. Some children in the study had co‐occurring diagnoses of ADHD or learning disorder, which were not part of the exclusion criteria. Additionally, some of these children were taking medications commonly used for ADHD, such as biphentin, concerta, adderall, vyvanse, and fluxetine. Both the treatment and waitlist groups also had children who received rehabilitation services including occupational therapy, physiotherapy, and speech therapy. However, children in the treatment group were asked to pause occupational therapy during the study. Table S3 reports co‐occurring conditions and interventions of each group. The groups did not show significant differences in terms of co‐occurring diagnoses, medication use, and rehabilitative interventions.

### Pretest‐posttest analysis

Results from the pre‐post analysis showed a statistically significant (*p* < 0.013) improvement in all outcome variables, including self‐rated performance and satisfaction of motor goals on the COPM, movement quality on the PQRS, and motor proficiency on the BOT‐2 (Table 2).

**TABLE 2:** Outcomes before and after CO‐OP intervention: pretest‐posttest analysis (*n* = 26).

Variable	Pretest median (IQR)	Posttest median (IQR)	95% CI difference	*p*	Effect size
COPM_Performance_	3.66 (2.75)	8.16 (2.33)	3.05–4.68	<0.001^a^	0.86
COPM_Satisfaction_	3.83 (2.46)	8.24 (2.67)	2.95–4.78	<0.001^a^	0.87
PQRS	3.33 (2.08)	6.00 (2.33)	1.93–3.04	<0.001^a^	0.85
BOT‐2 (percentile)	10 (12)	14 (15)	1.25–7.59	0.003^a^	0.58

Abbreviations: BOT‐2, Bruininks‐Oseretsky Test of Motor Proficiency, Second Edition; CI, confidence interval; CO‐OP, Cognitive Orientation to daily Occupational Performance; COPM, Canadian Occupational Performance Measure; IQR, interquartile range; PQRS, Performance Quality Rating Scale.

^a^ Bonferroni‐corrected *p* < 0.013.

Results demonstrated a clinically significant (2 points or more) increase in self‐perceived motor performance and satisfaction respectively for 92% and 85% of children. The distribution method indicates that a change score of 1.5 or more points in motor performance and a change score of 1.69 or more points in satisfaction are considered minimal clinically important differences, further confirming clinically significant improvements for most children; all children showed positive changes at the level of the standard error of measurement (Table S4). Both performance and satisfaction demonstrated large effect sizes (0.86, 95% confidence interval [CI]: 3.05–4.68 and 0.87, 95% CI: 2.95–4.78), indicating substantial and meaningful improvements.

Half of the children showed clinically significant improvement in movement quality as evidenced by a 3‐point change score on the PQRS. The effect size of 0.85 (95% CI: 1.93–3.04) indicates a large and meaningful effect, highlighting notable improvements achieved with the CO‐OP intervention.

The results showed a significant improvement in BOT‐2 scores, suggesting positive changes in motor proficiency. Although the effect size of 0.58 (95% CI: 1.25–7.59) represents a moderate effect, the result is not clinically meaningful.

To further confirm our findings, we compared the treatment group to the waitlist group before and after intervention (Table S2). The groups did not differ significantly before intervention, except that the waitlist group scored significantly higher on the PQRS at pretest. This significant difference was also observed at posttest, but the difference scores (range of improvement) were similar between groups.

### Follow‐up analysis

The results from the follow‐up analysis for the treatment group (Table 3) showed significant and meaningful improvements in self‐rated performance (*p* < 0.001, W = 0.69) and satisfaction (*p* < 0.001, W = 0.72) of motor goals on the COPM. Although there was a slight decline in scores at the follow‐up assessment compared to the posttest, the overall improvements from the pretest were still evident.

**TABLE 3:** Follow‐up analysis (*n* = 13).

Variable	Pretest median (IQR)	Posttest median (IQR)	Follow‐up median (IQR)	*p*	Effect size
COPM_Performance_	3.83 (2.83)	7.66 (2.33)	7.33 (2.50)	<0.001^a^	0.69
COPM_Satisfaction_	3.33 (3.25)	8.00 (3.50)	7.00 (3.33)	<0.001^a^	0.72
PQRS	2.66 (1.50)	5.33 (3.01)	5.33 (2.67)	<0.001^a^	0.65
BOT‐2 (percentile)	12 (12)	14 (16)	8 (13)	0.15	0.14

Abbreviations: BOT‐2, Bruininks‐Oseretsky Test of Motor Proficiency, Second Edition; COPM, Canadian Occupational Performance Measure; IQR, interquartile range; PQRS, Performance Quality Rating Scale.

^a^ Bonferroni‐corrected *p* < 0.013.

Furthermore, the motor quality measured by the PQRS also exhibited significant improvement post‐intervention (*p* < 0.001, r = 0.62). Similar to the COPM variables, there was a minor decrease in scores at the follow‐up, but the overall improvement from the pretest was still maintained.

In contrast, the BOT‐2 variable did not show significant changes over time (*p* = 0.15). The effect size was small (W = 0.14), suggesting minimal or insignificant changes in motor proficiency over time. Therefore, as expected, it appears that the intervention did not have a significant impact on motor proficiency as measured by the BOT‐2.

## DISCUSSION

Our results indicate that the CO‐OP intervention is effective for autistic children with DCD (without intellectual disability). Overall, the findings show significant improvements in self‐rated performance and satisfaction in motor‐related goals, as assessed by the COPM, as well as improved motor quality according to blinded therapist ratings on the PQRS. The improvement in motor proficiency measured by BOT‐2 was statistically significant, and the effect size suggests a moderate improvement compared to other outcomes.

Despite a slight decline in motor performance, motor satisfaction, and motor performance quality scores from posttest to follow‐up, the improvements observed from the pretest to follow‐up remained clinically and statistically significant. The slight decrease in BOT‐2 scores from the posttest to follow‐up suggests no significant loss in motor proficiency over this time.

Our results are consistent with our previous RCT examining the effectiveness of CO‐OP in children with DCD, with and without co‐occurring ADHD.^14^ Children with DCD and DCD + ADHD showed significant improvements in COPM and PQRS scores, with only small to moderate gains on the BOT‐2. At follow‐up, gains in motor goals and movement quality were maintained, but only children with DCD showed transferability to overall motor proficiency as measured by BOT‐2. Children with DCD + ADHD did not demonstrate transfer, nor did autistic children with DCD in the current study. In addition to ASD, over half of the sample (62%) had co‐occurring ADHD. These findings suggest that perhaps the impairments associated with co‐occurring conditions may impact the ability to transfer to other motor skills. Alternatively, the BOT‐2 may not be the most appropriate measure of transferability. Other research has measured generalization and transfer by incorporating an evaluation of an additional untrained task.^29^, ^30^ This entails a child setting four goals before CO‐OP sessions, with one goal designated as untrained to assess inter‐task transfer.

We acknowledge that this study has some limitations. First, given the cognitive nature of the CO‐OP intervention, we limited our sample to autistic participants without intellectual disability and those who communicated through verbal conversation. As such, results may not be generalizable to autistic children with intellectual disability and/or those who use different ways to communicate. Second, our sample size was smaller than expected, in part because of the global COVID‐19 pandemic, which led to a decrease in the power of the study from over 80% to 70%. An RCT with larger and more diverse samples is needed to validate these findings and enhance our understanding of motor interventions for autistic children with co‐occurring DCD. Third, it is important to note that while children in the study did not receive other motor‐based interventions, they may have received therapies for other areas such as social and communication skills. These additional therapies could have influenced the outcomes being measured. To address this potential confounder, we diligently documented all interventions received by each participant during the study and considered their potential influence on the results. Although we were not able to address this issue statistically, we had sufficient data to discuss this concern in the context of our findings. For example, we observed that two participants received therapies to improve their writing skills and bike riding abilities; however, these goals were not selected as specific targets of therapy for the study. Lastly, a limitation of this study concerns the sequential nature of baseline assessments, with the second group undergoing assessments after a 3‐month waiting period. While this approach was necessary to prevent potential interference with the intervention effects, it resulted in a lack of simultaneous baseline measurements for both groups. This limitation may have impacted the ability to control for natural changes over time and fully isolate the effects of the intervention.

Despite these limitations, this is the largest study to date to show that CO‐OP is effective in helping autistic children (without intellectual disability) learn motor skills. RCTs with larger samples and the inclusion of untrained tasks to better measure transfer are needed to confirm these findings. Results from this study provide promising evidence to support changes in clinical practice to include CO‐OP in treatment planning for autistic children to improve their motor and functional outcomes.

## CONFLICT OF INTEREST STATEMENT

The authors have no conflicts of interest to declare.

## Supporting information


**Figure S1:** Study design.

**Figure S2:** Consolidated Standards of Reporting Trials flowchart.

**Table S1:** Type and frequency of selected goals.

**Table S2:** Outcomes before and after CO‐OP intervention by group.

**Table S3:** Comparison of treatment and waitlist groups.

**Table S4:** Calculating minimal clinically important difference.

## Data Availability

The data that support the findings of this study are available from the corresponding author upon request.
