# Supplementary material for: Effectiveness of Cognitive Orientation to daily Occupational Performance for autistic children with developmental coordination disorder
Source: Dev Med Child Neurol. 2024 Aug 14;67(2):216–22. doi: 10.1111/dmcn.16058 (PMC11695746; doi:10.1111/dmcn.16058)
Supplement: Supplementary file 1 — Figure S1: Study design. [file DMCN-67-216-s002.docx]

**Baseline**

**3 Months**

**6 Months**

**Figure S1:** Study design

Abbreviations: ASD, autism spectrum disorder; ASSQ, Autism Spectrum Screening Questionnaire; BOT-2, Bruininks-Oseretsky Test of Motor Proficiency–2nd edition; CO-OP, cognitive orientation to occupational performance; COPM, Canadian Occupational Performance Measure; DCD, developmental coordination disorder; DCDQ, Developmental Coordination Disorder Questionnaire; MABC-2, Movement Assessment Battery for Children–2nd edition; PQRS, Performance Quality Rating Scale.

**MABC-2**

**DCDQ**

**ASSQ**

**Conners 3 ADHD Index**

Pretest - Posttest

Pretest - Posttest

Follow-up

**COPM**

**PQRS**

**BOT-2**

**COPM**

**PQRS**

**BOT-2**

**COPM**

**PQRS**

**BOT-2**

**Treatment**

**DCD+ASD (N=13)**

**Waitlist**

**DCD+ASD**

**(N=13)**

**DCD+ASD**

**N=26**

**Screening**

**Randomization**

**No Intervention**

**No Intervention**

**CO-OP**

**COPM**

**PQRS**

**BOT-2**

**COPM**

**PQRS**

**BOT-2**

**CO-OP**
