# Supplementary figures and images for: Effectiveness of Cognitive Orientation to daily Occupational Performance for autistic children with developmental coordination disorder
Source: Dev Med Child Neurol. 2024 Aug 14;67(2):216–22. doi: 10.1111/dmcn.16058 (PMC11695746; doi:10.1111/dmcn.16058)

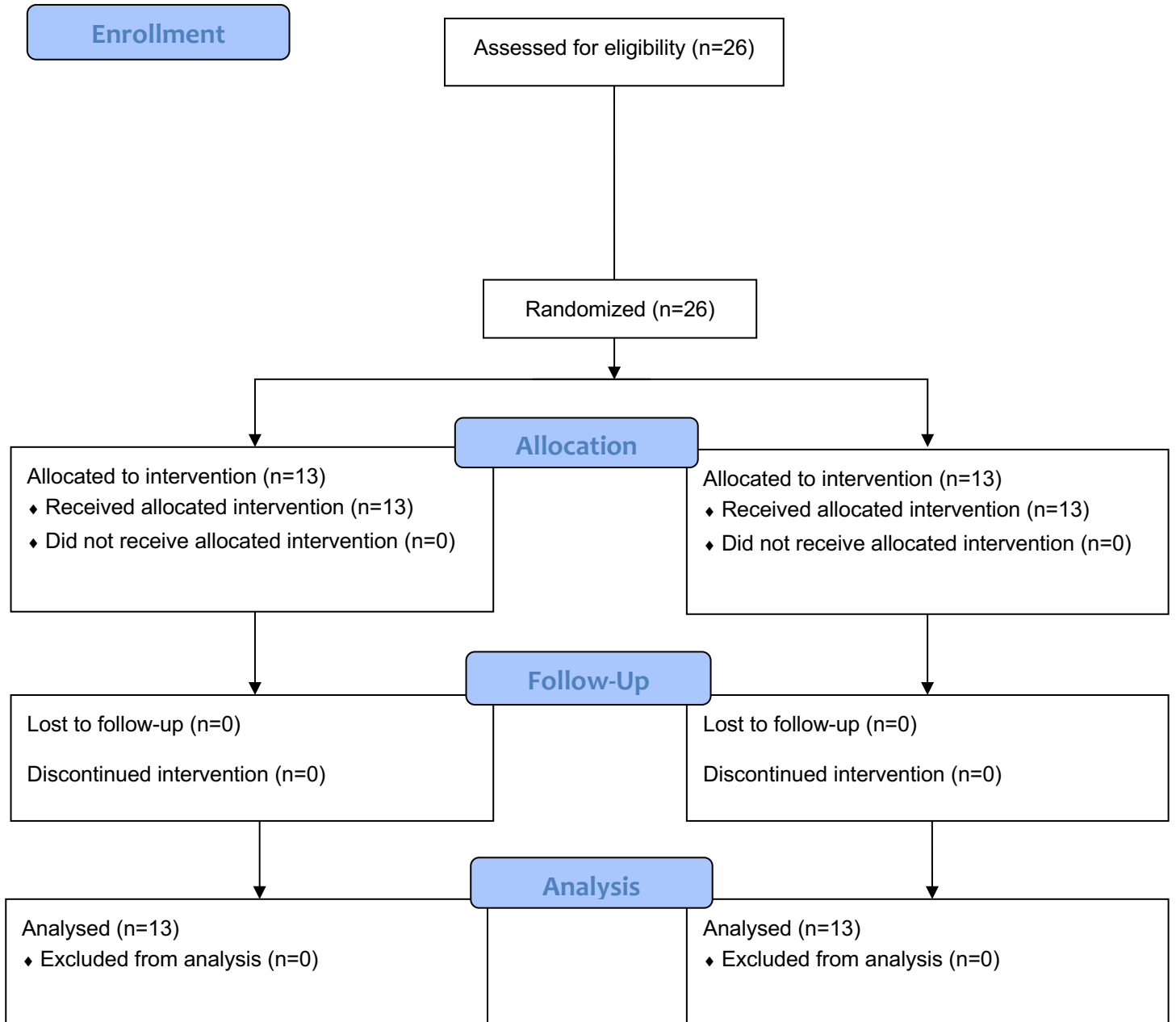

Figure S2: Consolidated Standards of Reporting Trials flow diagram

Supplement: Supplementary file 2 — Figure S2: Consolidated Standards of Reporting Trials flowchart. [file DMCN-67-216-s005.pdf]
