# Supplementary material for: Effectiveness of Cognitive Orientation to daily Occupational Performance for autistic children with developmental coordination disorder
Source: Dev Med Child Neurol. 2024 Aug 14;67(2):216–22. doi: 10.1111/dmcn.16058 (PMC11695746; doi:10.1111/dmcn.16058)
Supplement: Supplementary file 3 — Table S1: Type and frequency of selected goals. [file DMCN-67-216-s006.pdf]

Supplementary Table 1. Type and Frequency of Selected Goals

	Goals	Treatment (n=13)	Waitlist (n=13)
**Self-care**	Tying shoes	5	8
	Using utensil to cut vegetables/fruit	2	5
	Using utensil to eat	2	1
**School**	Printing	4	4
	Handwriting	4	2
	Typing	1	1
	Other	0	1
**Leisure**	Badminton	3	0
	Basketball	1	1
	Baseball	0	1
	Volleyball	1	1
	Soccer/Football	3	2
	Throwing and catching	5	4
	Skipping a rope	3	1
	Running	1	3
	Frisbee	0	2
	Riding a bike	0	1
	Other	4	1
