# Supplementary material for: Effectiveness of Cognitive Orientation to daily Occupational Performance for autistic children with developmental coordination disorder
Source: Dev Med Child Neurol. 2024 Aug 14;67(2):216–22. doi: 10.1111/dmcn.16058 (PMC11695746; doi:10.1111/dmcn.16058)
Supplement: Supplementary file 4 — Table S2: Outcomes before and after CO‐OP intervention by group. [file DMCN-67-216-s001.pdf]

Supplementary Table 2. **Outcomes before and after CO-OP intervention by group**

	Treatment (n=13)					Waitlist (n=13)				
**Variable**	**Pretest Median (IQR)**	**Posttest Median (IQR)**	**P-value**	**Effect size**	**95% CI Difference**	**Pretest Median (IQR)**	**Posttest Median (IQR)**	**P-value**	**Effect size**	**95% CI Difference**
$COPM_{Performance}$	3.66 (2.83)	7.66 (2.33)	0.001[a]	0.87	6.08 - 9.24	3.66 (2.33)	8.33 (1.83)	0.003[a]	0.82	6.99 - 9.67
$COPM_{Satisfaction}$	3.33 (3.25)	8.0 (3.50)	0.001[a]	0.87	6.06 - 9.94	4.0 (2.50)	8.33 (2.5)	0.001[a]	0.88	6.59 - 10.07
PQRS	2.66 (1.5)	5.33 (3.01)	0.002[a]	0.84	3.19 - 7.47	4.00 (2.3)	7.00 (2.0)	0.001[a]	0.87	5.49 - 8.51
BOT-2 (percentile)	12 (12)	14 (16)	0.046	0.55	3.65 - 24.35	8 (15)	14 (17)	0.026	0.61	1.95 - 26.05

BOT-2: Bruininks-Oseretsky Test of Motor Proficiency-2nd edition; CI: confidence interval; COPM: Canadian Occupational Performance Measure; IQR: inter-quartile range; PQRS: Performance Quality Rating Scale.

[a]Bonferroni-corrected $p<0.0125$.
