# Supplementary material for: Effectiveness of Cognitive Orientation to daily Occupational Performance for autistic children with developmental coordination disorder
Source: Dev Med Child Neurol. 2024 Aug 14;67(2):216–22. doi: 10.1111/dmcn.16058 (PMC11695746; doi:10.1111/dmcn.16058)
Supplement: Supplementary file 5 — Table S3: Comparison of treatment and waitlist groups. [file DMCN-67-216-s003.pdf]

Supplementary Table 3. Comparison of Treatment and Waitlist Groups

Variables	Treatment (n=13)	Waitlist (n=13)	P-value
**Pretest scores [median (IQR)]**			
$COPM_{Performance}$	3.83 (2.83)	3.66 (2.33)	0.29
$COPM_{Satisfaction}$	3.33 (3.25)	4.00 (2.50)	0.28
PQRS	2.66 (1.50)	4.00 (2.33)	0.002[a]
BOT-2 (percentile)	12 (12)	8 (15)	0.95
**Posttest scores [median (IQR)]**			
$COPM_{Performance}$	7.66 (2.33)	8.33 (1.83)	0.70
$COPM_{Satisfaction}$	8.00 (3.50)	8.33 (2.50)	0.75
PQRS	5.33 (3.01)	7.00 (2.00)	0.009[a]
BOT-2 (percentile)	14 (16)	14 (17)	0.64
**Difference scores [median (IQR)]**			
$COPM_{Performance}$	3.66 (2.58)	4.00 (3.00)	0.88
$COPM_{Satisfaction}$	3.32 (4.25)	3 (3.16)	0.76
PQRS	1.67 (2.51)	2.34 (1.67)	0.55
BOT-2 (percentile)	2 (7.5)	3 (6.5)	0.68
**Co-occurring conditions and interventions [N (%)]**			
Co-occurring ADHD	8 (62)	8 (62)	1.00
Co-occurring learning disorder	2 (15)	4 (31)	0.07
Co-occurring other diagnosis	3 (23)	2 (15)	0.34

Physical therapy intervention	1 (8)	2 (15)	0.23
Occupational therapy intervention	0 (0)	2 (15)	0.001[a]
Speech therapy intervention	5 (38)	3 (23)	0.11

ADHD, attention deficit hyperactivity disorder; BOT-2, Bruininks-Osteretsky Test of Motor Proficiency – 2nd edition; COPM, Canadian Occupational Performance Measure; IQR, interquartile range; PQRS, Performance Quality Rating Scale

[a]Statistically significant result.
