# Supplementary material for: Effectiveness of Cognitive Orientation to daily Occupational Performance for autistic children with developmental coordination disorder
Source: Dev Med Child Neurol. 2024 Aug 14;67(2):216–22. doi: 10.1111/dmcn.16058 (PMC11695746; doi:10.1111/dmcn.16058)
Supplement: Supplementary file 6 — Table S4: Calculating minimal clinically important difference. [file DMCN-67-216-s004.pdf]

Supplementary Table 4. Calculating minimal clinically important difference

Variables	Mean (SD)	SEM	MCID
Difference scores (posttest-pretest)			
$COPM_{Performance}$	3.87 (2.02)	0.396	1.51
$COPM_{Satisfaction}$	3.87 (2.26)	0.443	1.69

COPM, Canadian Occupational Performance Measure; MCID, Minimal Clinically Important Difference (0.75xSD); SD, standard deviation; SEM, Standard Error of Measurement
